# Immunocytoprotection after reperfusion with Kv1.3 inhibitors has an extended treatment window for ischemic stroke

**DOI:** 10.3389/fphar.2023.1190476

**Published:** 2023-04-25

**Authors:** Ruth D. Lee, Yi-Je Chen, Latika Singh, Hai M. Nguyen, Heike Wulff

**Affiliations:** ^1^ Department of Pharmacology, School of Medicine, University of California, Davis, Davis, CA, United States; ^2^ Animal Models Core, Department of Pharmacology, School of Medicine, University of California, Davis, Davis, CA, United States

**Keywords:** Kv1.3, microglia activation, ischemic stroke, neuroinflammation, potassium channel, PAP-1, ShK toxin

## Abstract

**Introduction:** Mechanical thrombectomy has improved treatment options and outcomes for acute ischemic stroke with large artery occlusion. However, as the time window of endovascular thrombectomy is extended there is an increasing need to develop immunocytoprotective therapies that can reduce inflammation in the penumbra and prevent reperfusion injury. We previously demonstrated, that by reducing neuroinflammation, K_V_1.3 inhibitors can improve outcomes not only in young male rodents but also in female and aged animals. To further explore the therapeutic potential of K_V_1.3 inhibitors for stroke therapy, we here directly compared a peptidic and a small molecule K_V_1.3 blocker and asked whether K_V_1.3 inhibition would still be beneficial when started at 72 hours after reperfusion.

**Methods:** Transient middle cerebral artery occlusion (tMCAO, 90-min) was induced in male Wistar rats and neurological deficit assessed daily. On day-8 infarction was determined by T2-weighted MRI and inflammatory marker expression in the brain by quantitative PCR. Potential interactions with tissue plasminogen activator (tPA) were evaluated *in-vitro* with a chromogenic assay.

**Results:** In a direct comparison with administration started at 2 hours after reperfusion, the small molecule PAP-1 significantly improved outcomes on day-8, while the peptide ShK-223 failed to reduce infarction and neurological deficits despite reducing inflammatory marker expression. PAP-1 still provided benefits when started 72 hours after reperfusion. PAP-1 does not reduce the proteolytic activity of tPA.

**Discussion:** Our studies suggest that K_V_1.3 inhibition for immunocytoprotection after ischemic stroke has a wide therapeutic window for salvaging the inflammatory penumbra and requires brain-penetrant small molecules.

## 1 Introduction

The goal of acute ischemic stroke therapy is to minimize damage by restoring blood flow to ischemic brain areas as soon as possible while minimizing reperfusion injury. Since the first endovascular device was approved by the FDA in 2004, door-to-reperfusion times have been slowly improving, with an increasing number of patients receiving and benefiting from Endovascular Therapy (EVT) (Goyal et al., 2016; Smith and Furlan, 2016). For acute ischemic stroke patients with a proximal cerebral arterial occlusion and salvageable brain tissue seen on Computerized Tomography (CT) imaging, EVT has been shown to be safe and efficacious within 6 h after symptom onset (Campbell et al., 2015; Goyal et al., 2015). And, for a subset of patients who have severe symptom deficits despite a relatively small infarct volume, several studies have extended this treatment window to 24 h and beyond (Nogueira et al., 2018).

Even though patient outcomes are significantly improved when EVT is initiated as soon as possible (Saver et al., 2016), door-to-reperfusion times can vary widely, depending on factors like patient education about stroke, and distance from and access to EVT-capable hospitals (Saver et al., 2016). Today’s acute stroke algorithm entails transporting patients to the nearest hospital, capable of providing diagnostic imaging and intravenous tissue-type plasminogen activator (IV-tPA), but not necessarily EVT; only about 20% of stroke patients in the USA are within fifteen minutes of transportation time to an EVT-capable hospital (Saver et al., 2016). Thus, most patients must be subsequently transferred to an EVT-capable facility, with an average symptom onset-to-procedure time of 4.3 h (Saver et al., 2016). This broad range of door-to-reperfusion times can result in an enlarging ischemic core (Gauberti et al., 2016; Savitz et al., 2017), which in turn, may explain why some patients have such poor outcomes after thrombectomy. Most of the injury in the ischemic core results from the lack of blood flow itself, which is why it is salvageable by reperfusion via thrombectomy, but with time, this core can enlarge into the ischemic or “inflammatory” penumbra (Gauberti et al., 2016), which are areas at risk of damage by immune cells like microglia and infiltrating neutrophiles, macrophages and T-cells (Weinstein et al., 2010; Iadecola and Anrather, 2011; Chamorro et al., 2012). Because there is a strong neuroinflammatory component to the penumbra, lesion growth, and the poor neurological outcomes associated with it despite successful thrombectomy (Zhou et al., 2023), may be preventable by immunomodulatory drugs providing what has been termed “immunocytoprotection” (Gauberti et al., 2016; Savitz et al., 2017). A recent study reported radiological observed reperfusion injury (RORI) in 47% of stroke patients after mechanical thrombectomy (Zhou et al., 2023).

Here, we focus on one possible target of immunomodulation for ischemic stroke. We previously showed that blocking the voltage-gated potassium channel K_V_1.3, which is expressed on microglia and T cells, with our small molecule drug, PAP-1, results in reduced neuroinflammation without compromising the beneficial functions of the immune system such as phagocytosis of neuronal debris by microglia in two rodent models of ischemic stroke (Chen et al., 2018; Chen et al., 2021). Using middle cerebral artery occlusion (MCAO) with reperfusion in both mice (60 min occlusion) and rats (90 min occlusion), we demonstrated that K_V_1.3 inhibition with PAP-1 reduces infarct size and improves neurological deficits on day-8 in young adult male mice and rats when compound administration was started 12 h after reperfusion (Chen et al., 2018). We subsequently further validated K_V_1.3 as a target for immunocytoprotection in ischemic stroke by demonstrating that pharmacological K_V_1.3 blockade and genetic K_V_1.3 deletion benefits young (16-week-old) and aged (80-week-old) mice of both sexes (Chen et al., 2021). Here, we explore the therapeutic window of PAP-1 and assess the degree of immunocytoprotection PAP-1 confers when given earlier and later than our previous 12-h timepoint, at 2 h and 3 days after MCAO in rats. We also evaluated a peptidic K_V_1.3 blocker in order to compare peripherally restricted K_V_1.3 inhibition with the brain-penetrant K_V_1.3 blocker PAP-1.

## 2 Materials and methods

### 2.1 Middle cerebral artery occlusion surgeries

This study was approved by the University of California, Davis, Animal Use and Care Committee and conducted in accordance with the guidelines of Animal Use and Care of the National Institutes of Health, the University of California, Davis for survival surgery, and the IMPROVE guidelines for ischemic stroke in rodents (Percie du Sert et al., 2017). Adult male Wistar IGS rats (Strain 003 WISTAR) weighing 160–180 g were purchased from Charles River (Wilmington, MA, USA), acclimatized to the vivarium for 7–10 days and used for surgery when they weighed 250–300 g. Rats were anesthetized using box induction with 5% isoflurane and then maintained on 0.5%–1.5% isoflurane in medical grade oxygen via a facemask. To assure consistent reduction of cerebral blood flow (CBF) throughout the procedure, a small area of skull was shaved, and the Laser Doppler probe (Moor Instruments, Wilmington, DE, USA) affixed to the surface of the skull with a hand-made adapter 5 mm lateral to the central fusion line and 2.5 mm posterior to bregma after the skull bone had been thinned with a drill (5 mm diameter). Instant adhesive and dental cement were applied to the base and around the edges of the small plastic adapter to hold the Doppler probe. The adapter with the attached probe remained in place throughout the MCAO surgery to confirm continuous occlusion and later the establishment of reperfusion. Focal cerebral ischemia was then induced by occlusion of the left middle cerebral artery (MCA) according to Longa et al. (1989). Briefly, the left common carotid artery was surgically exposed, the external carotid artery was ligated distally from the common carotid artery, and a silicone rubber-coated nylon monofilament with a tip diameter of 0.43 ± 0.02 mm (Doccol Corp., Redlands, CA, USA) was inserted into the external carotid artery and advanced into the internal carotid artery to block the origin of the MCA (when maximum CBF reduction observed). The filament was kept in place for 90 min and then withdrawn to restore blood supply. Animals received subcutaneous Buprenex at 0.02 mg/kg every 12 h to limit postsurgical pain for 24 h after surgery. For sham surgeries, the filament was placed into the external carotid artery but not advanced into the internal carotid artery.

The survival rate for MCAO surgeries was 92%. Animals where CBF was not reduced by at least 70% and which did not display any obvious neurological deficit 2 h after reperfusion were excluded. The average CBF reduction in this study was 76.3% ± 5.8%. Animals that met these inclusion criteria were assigned to treatment or vehicle groups based on a computer-generated randomization scheme. Out of the animals surviving to day-8 (52 out of 56), 4 animals were excluded due to insufficient occlusion and insufficient neurological deficit.

### 2.2 Drug treatment

**FIGURE 1:**
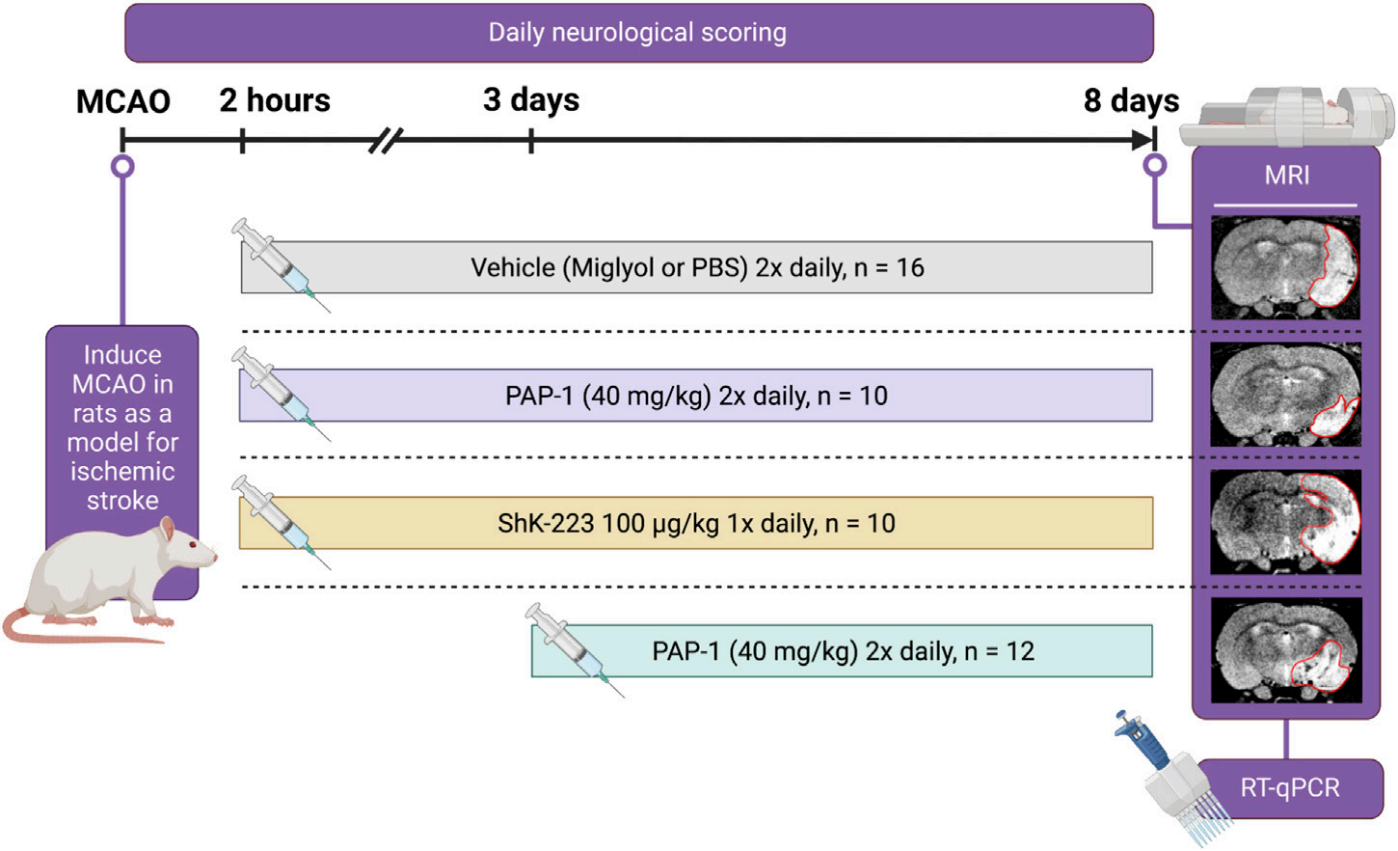
Experimental Paradigm. Cartoon showing the experimental design, the timeline, and the treatment groups.

Starting at 2 h or 3 days post-MCAO, rats received either miglyol-812 neutral oil (Caprylic/Capric Triglyceride, Spectrum Chemicals), saline, ShK-223 at 100 mg/kg once daily, or PAP-1 at 40 mg/kg intraperitoneally twice daily until sacrifice on day-8 (Figure 1). Miglyol 812 is a low viscosity oily vehicle, which is widely used as a pharmaceutical excipient and well tolerated for i. p. or oral administration. It can be autoclaved and allows for the dissolution of lipophilic compounds like PAP-1 at high concentrations (20 mg/mL for this study). PAP-1 was synthesized in our laboratory and chemical identity and purity (>98%) confirmed by ^1^H and ^13^C-NMR and HPLC (Schmitz et al., 2005). ShK-223, a highly Kv1.3-selective derivative of the sea anemone *Stichodactyla helianthus* toxin peptide ShK (Pennington et al., 2015) was synthesized at AmbioPharm Inc. (North Augusta, SC).

### 2.3 Neurological scoring

Neurological deficits were scored according to a 14-score test tactile and proprioceptive limb-placing test (De Ryck et al., 1989) as follows: proprioception, forward extension, lateral abduction, and adduction were tested with vision or tactile stimuli (lower score for more severe neurological deficits). For visual limb placing, rats were held and slowly moved forward or lateral toward the top of a table. Normal rats placed both forepaws on the tabletop. Tactile forward and lateral limb placing were tested by lightly contacting the table edge with the dorsal or lateral surface of a rat’s paw while avoiding whisker contact and covering the eyes to avoid vision. For proprioceptive hindlimb placing, each rat was pushed along the edge of an elevated platform to test proprioceptive hindlimb adduction. The paw was pulled down and away from the platform edge, and the ability to retrieve and place the paw on the table surface upon sudden release was assessed. For each test, limb-placing scores were 0 = no placing; 1 = incomplete and delayed (>2 s) placing; or 2 = immediate and complete placing. For each body side, the maximum summed tactile and proprioceptive limb-placing score, including the platform test, was 14.

The investigator performing the drug administration, neurological scoring, and analyses was blinded to the treatments.

### 2.4 Assessment of infarct area with MRI

Infarct volume on day-8 was evaluated with T2-weighted MRI imaging in the Nuclear Magnetic Resonance (NMR) Facility at UC Davis. MRI was performed with a 7T (300 MHz) Bruker Biospec MR system running ParaVision version 5. The RF coil was Bruker’s standard 42 mm ID rat resonator. Animals were anesthetized with isoflurane and placed under a heated circulating water blanket (37°C) to maintain body temperature. Fast spin echo (FSE) imaging, also known as RARE (Rapid Acquisition with Relaxation Enhancement), sequence was used to acquire tri-pilot geometry reference images. The multi-slice multi-echo (MSME) sequence (TE:56 ms; TR: 1681.2 ms) was used to acquire images of 12 coronal sections with 1 mm thickness from the junction of olfactory bulb and cortex. The infarct area was analyzed with Adobe Photoshop Elements by an investigator blinded to the treatments. The percentage of infarcted area was calculated with the equation: (Infarct Area ÷ Ipsilateral Hemisphere Area) × 100% MRI.

### 2.5 Statistics

Based on our previous work with PAP-1 in MCAO (Chen et al., 2018), sample size was powered to detect a reduction of 40% in mean percentage infarct area with 80% power. Statistical analysis comparing two groups was done using unpaired *t*-test. Comparison of three groups was done using one-way ANOVA in Prism, with *post hoc* pairwise comparison of the treatment groups with Tukey’s method (Schlattmann and Dirnagl, 2010). *p* < 0.05 was used as the level of significance; *p < 0.05, **p < 0.01, and ***p < 0.001. All data are given as mean ± S.E.M.

### 2.6 RT-qPCR

After the MRI was completed, rats were euthanized with an overdose of isoflurane, and brains were harvested. The brains were split into ipsilateral and contralateral hemispheres and were immediately frozen with liquid N_2_. Brain hemispheres were homogenized in mortar and pestles with liquid N_2_. RNA was subsequently extracted with Trizol (ThermoFisher, Catalog # 15596026) according to the manufacturer’s protocol. RNA purity and concentration were assessed using a Nanodrop Spectrophotometer ND-1000 (Marshall Scientific). The 260 nm/280 nm absorption ratios for the samples ranged from 1.90 to 2.00. Subsequently, a cDNA library was made with a High-Capacity cDNA Reverse Transcription Kit (ThermoFisher Scientific, Catalog# 4374967), with 2 µg of total RNA per 20 uL reaction. Reactions were carried out in a PTC-200 Peltier Thermal Cycler; the conditions were as follows: 25°C for 10 min, 37°C for 120 min, 85°C for 5 min, with final storage at 4°C. RT-qPCR reactions were conducted in triplicate in an Applied Biosystems Viia7 Real-Time PCR System using Maxima SYBR Green/ROX qPCR Master Mix (ThermoFisher Scientific, Catalog #K022) and with the recommended three-step cycling protocol. RT-qPCR primer sequences are provided in Supplementary Table S1; The primers for rat *IL-2*, *IL-4*, *IL-10*, and *IL-17a* did not work as determined with concanavalin A stimulated rat splenocytes.

All CT values were normalized to the geometric mean of three housekeeping genes, β-*actin*, *hmbs*, and *ywhaz*, as recommended by Vandesompele et al. (2002). Fold changes were calculated by the ∆∆Ct method; relative mRNA levels were generated by normalizing to sham. The three housekeeping genes were selected from a panel of the 17 most commonly used housekeeping genes in the stroke field because they were the most stably expressed genes (with the lowest M values) in two age-matched male Wistar rats and two representative ipsilateral MCAO Wistar rats; this ranking was generated by a stepwise exclusion of the least stable genes, using the GeNorm analysis software (Vandesompele et al., 2002; Kang et al., 2018). We chose both normal brain and ipsilateral MCAO hemispheres for the housekeeping gene panel because we wanted genes that remained stable during stroke. Primers were purchased from BioRad as part of their PrimePCR plate and sequences are provided in Supplementary Table S2. Outliers were defined as datapoints that deviated more than 5 standard deviations from the mean and were removed (modified Grubb’s test). The remaining 612 data points were analyzed and are shown. Statistical analysis comparing the ipsilateral hemisphere of the various treatment groups to the ipsilateral hemisphere of the vehicle treated animals was performed in Prism using one-way ANOVA followed by Dunnett’s test to correct for multiple comparisons. All data are given as mean ± S.E.M.

### 2.7 tPA assay

The commercially available Tissue tPA Activity Assay Kit (Abcam, ab108905) was used to assess whether PAP-1 affects the ability of human tPA to cleave its substrate plasminogen (Lapchak and Boitano, 2014; Lapchak et al., 2017). All reactions were run in optically clear flat-bottom 96-well plates (Corning, Catalog #3904). Human plasminogen activator inhibitor-1 (PAI-1, Sigma-Aldrich CC4075) was used as a positive control. Human plasminogen, the chromogenic plasmin substrate, and human tPA were included in the Tissue tPA Activity Assay Kit (Abcam, ab108905). PBS with 10% human serum was used as the assay medium; PAP-1 was added at 500 nM and 5 μM with a final DMSO concentration of 0.1%. The reactions were pre-incubated at 37°C for 5 min prior to addition of the chromogenic substrate and placed into a SpectraMax M5 spectrophotometer heated to 37°C. Absorbances at 405 nm were measured every 30 s for 30 min.

## 3 Results

### 3.1 K_V_1.3 inhibition with the small molecule PAP-1 but not the peptide ShK-223 reduces infarction and improves neurological deficit after tMCAO in rats

K_V_1.3 can be targeted with small molecules or with biologics such as venom-derived peptides, antibodies and nanobodies. We here chose two K_V_1.3 blockers, our small molecule PAP-1 (Schmitz et al., 2005) and ShK-223 (Pennington et al., 2015), a more selective and stable analog of ShK-186 (Dalazatide) (Tarcha et al., 2012), as representatives of the two modalities for testing in MCAO in adult male Wistar rats. The doses of ShK-223 (IC_50_ 25 p.m.) and PAP-1 (IC_50_ for 2 nM) were chosen considering their Kv1.3 blocking potency and are based on previously reported efficacy in rat models of experimental autoimmune encephalomyelitis (EAE) or of ischemic stroke (Tarcha et al., 2012; Chen et al., 2018).

**FIGURE 2:**
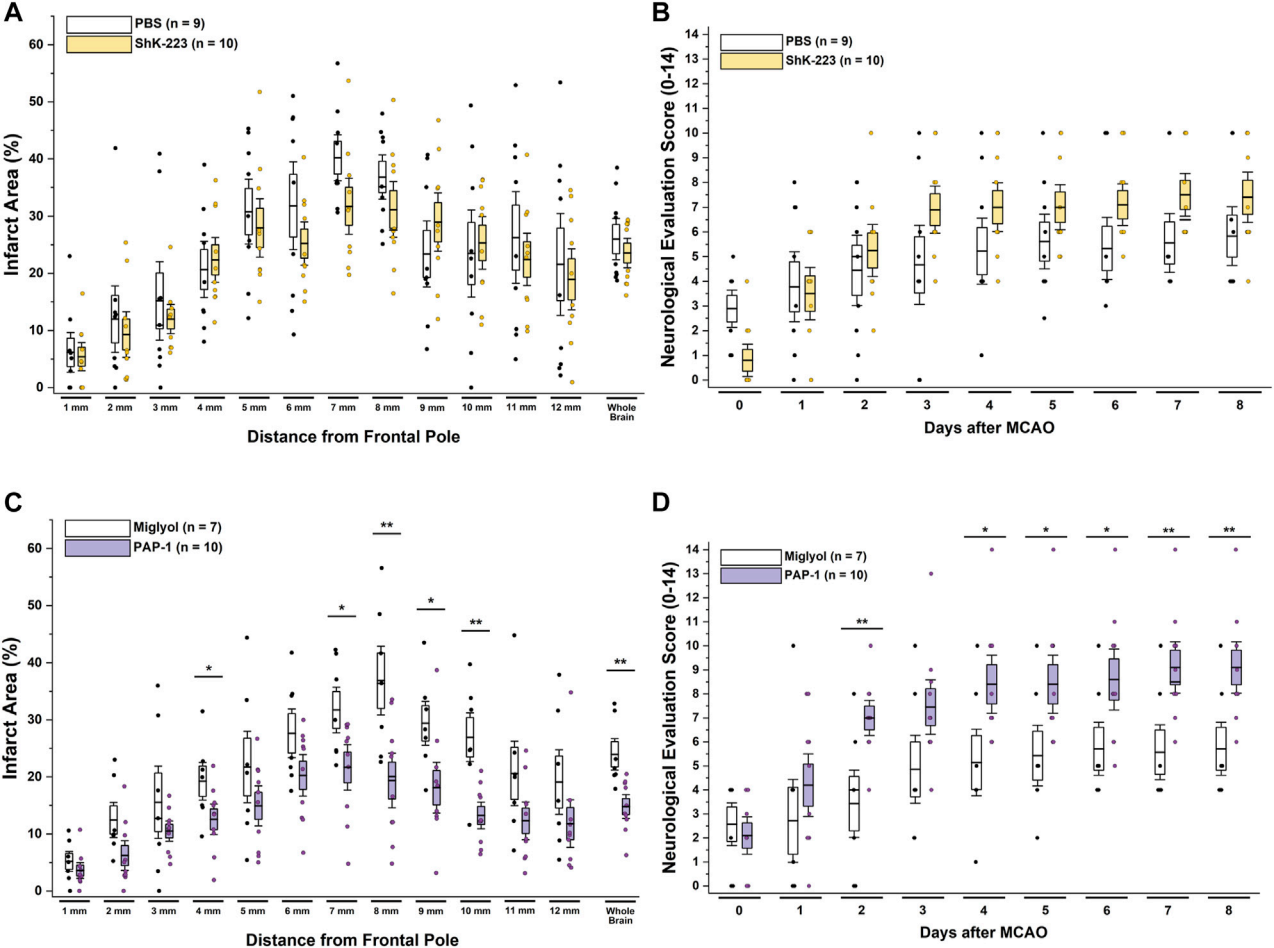
The K_V_1.3 blocker PAP-1 but not the peptide ShK-223 reduces infarction and improves neurological deficit in adult male rats. **(A)** Infarct area and **(B)** neurological deficit score in PBS (n = 9) compared to ShK-223 (100 mg/kg; n = 10) treated male Wistar rat. **(C)** Infarct area and **(D)** neurological deficit score in miglyol vehicle (n = 7) compared to PAP-1 (40 mg/kg; n = 10) treated male rats (*p* = 0.002 for infarct, *p* = 0.009 for NES on day-8). Data are shown as whisker plots with data from individual animals overlayed as scatter. The boxes show mean ± S.E.M; the whiskers show confidence intervals.

Following 90 min of occlusion of the MCA (76.3% ± 5.8% flow reduction) and subsequent reperfusion, rats were randomized and treated either with 100 mg/kg ShK-223, 40 mg/kg PAP-1 or the respective compound vehicles (PBS or miglyol) starting 2 h after reperfusion (Figure 1). The treatments were administered twice daily and continued until day-8, when infarct areas were measured by T2-weighted MRI and brains subsequently removed for qPCR analysis. Neurological deficit scoring using the De Ryck 14-score tactile and proprioceptive limb-placing test (De Ryck et al., 1989) was performed daily, starting 12 h after reperfusion. While ShK-223 did not reduce infarction (Figure 2A) or improve neurological deficit scores (Figure 2B) compared to vehicle-treated rats, PAP-1 treatment significantly reduced infarct area on day-8 (Figure 2C) and ameliorated neurological deficit starting from day-4 after MCAO (Figure 2D) when compared to its vehicle miglyol, which is a low viscosity triglyceride. Since we had used two different vehicles, we next compared the two vehicles to each other and found no differences in stroke outcomes (Supplementary Figures S1A, B). We, therefore, combined the two vehicles into one large vehicle group (n = 16) and compared it to ShK-223 and PAP-1 treated animals. This comparison using Turkey’s method for *post hoc* comparison, again demonstrated that the small molecule PAP-1 improved stroke outcomes, while the peptidic K_V_1.3 blocker ShK-223, did not significantly reduce infarction (Supplementary Figure S1C) or improved neurological deficit (Supplementary Figure S1D).

### 3.2 K_V_1.3 inhibition with PAP-1 still improves MCAO outcomes when administration is started 3 days after reperfusion

Following MCAO in rats and mice, hypertrophic, CD68-positive mononuclear phagocytes, arising from both activated microglia and infiltrating monocytes become abundant in the infarcted area and the penumbra between 18 and 96 h after an ischemic stroke and have been described to peak between 7–14 days (Campanella et al., 2002; Hu et al., 2012). Using immunohistochemistry and electrophysiology on acutely isolated CD11b^+^ cells we previously characterized the time course of K_V_1.3 expression on microglia/macrophages following ischemic stroke in mice. While K_V_1.3 currents were barely detectable on microglia isolated from normal brain or from the contralateral side, K_V_1.3 current amplitudes from CD11b^+^ cells from the infarcted hemisphere started to significantly increase at two and 5 days after MCAO and peaked around day-8 (Chen et al., 2018). Similar to mice, we here observed large, PAP-1-sensitive K_V_1.3 currents on Iba-1 and CD68^+^ microglia/macrophages acutely isolated from the infarcted area with CD11b-selective magnetic beads (Supplementary Figure S2). A significant percentage of the CD11b^+^ cells were clearly activated and stained positive for K_V_1.3, confirming our previous observations made in mice, here in rats.

**FIGURE 3:**
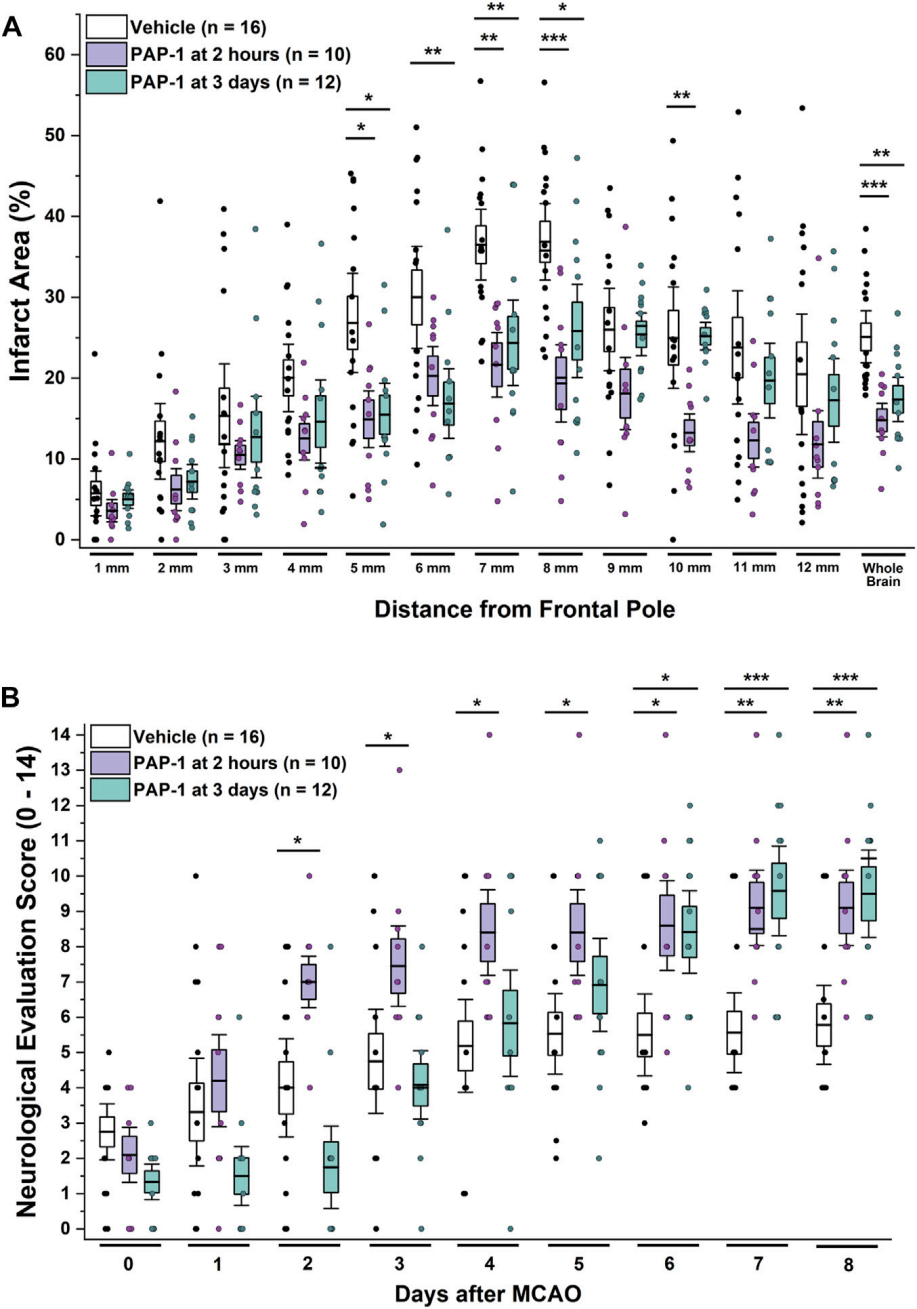
K_V_1.3 inhibition with PAP-1 still improves MCAO outcomes when administration is started 3 days after reperfusion. **(A)** Infarct area and **(B)** neurological deficit score in vehicle treated male Wistar rats (n = 16) compared to animals treated with 40 mg/kg PAP-1 started at 2 h (n = 10, *p* = 0.0004 for infarct, *p* = 0.005 for NES on day-8) or started at 3 days (n = 12, *p* = 0.005 for infarct, *p* = 0.0009 for NES on day-8) after reperfusion. Data are shown as whisker plots with data from individual animals overlayed as scatter. The boxes show mean ± S.E.M; the whiskers show confidence intervals. PAP-1 started at 2 h was not significantly different from PAP-1 started at 3 days.

Based on these time courses for microglia/macrophage activation and K_V_1.3 expression, we hypothesized that K_V_1.3 inhibition might still be beneficial when started as late as 3 days after ischemic stroke and therefore also treated a group of rats with 40 mg/kg of PAP-1 starting at 72 h after reperfusion. A comparison of these animals to vehicle-treated rats and rats that had started receiving PAP-1 at 2 h after reperfusion, revealed that delayed PAP-1 treatment was as effective as early PAP-1 treatment in reducing infarction as determined by T2-weighted MRI by day-8 after MCAO (Figure 3A). As expected, neurological deficit scoring was initially not altered in comparison to vehicle but started to be significantly improved by day-6 and was similar to early PAP-1 by day-8 (Figure 3B). These findings suggest that PAP-1 is able to salvage the “inflammatory” penumbra (Gauberti et al., 2016).

### 3.3 Early and late administration of K_V_1.3 inhibitors reduces inflammatory marker expression in the infarcted hemisphere

**FIGURE 4:**
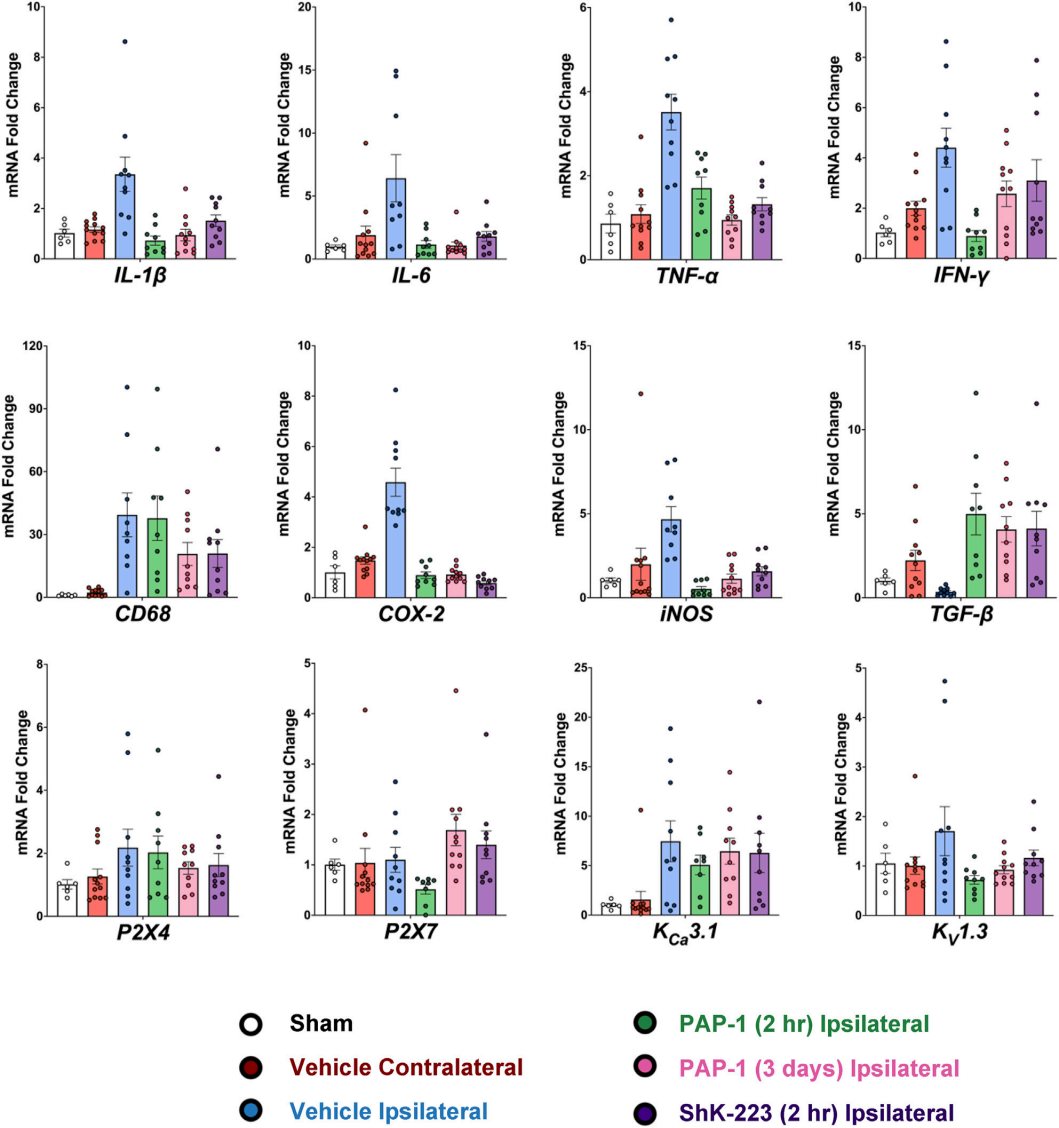
Blocking K_V_1.3 with a brain-penetrant small molecule, PAP-1, and brain-impermeable peptide, ShK-223, both reduce pro-inflammatory cytokines *in-vivo*, as measured by RT-qPCR. mRNA expression levels are reported as a fold-change respective to the ipsilateral hemisphere of vehicle-treated MCAO mice. Values were normalized to sham rat brains and to the geometric mean of three housekeeping genes, *β-actin*, *ywhaz*, and *hmbs*. Data was analyzed with one-way ANOVA followed by Dunnett’s test and are shown as mean ± S.E.M; the whiskers show confidence intervals. For full statistics see Supplementary Table S3.

Following the MRI on day-8, brains from all animals were removed for RT-qPCR analysis. As recommended by Vandesompele *et al.*, CT values were normalized to three housekeeping genes (Vandesompele et al., 2002), *β-actin*, *hmbs*, and *ywhaz,* which were selected from a panel of the 17 most commonly used housekeeping genes in the stroke field based on their stable expression when comparing age matched male Wistar rats and ipsilateral MCAO tissue (see Methods). Relative mRNA levels of all analyzed markers were normalizing to sham. MCAO significantly increased expression of inflammatory cytokines (*IL-1β*, *IL-6*, *TNF-α* and *IFN-γ*), the phagocyte marker CD68, and the enzymes *COX-2* and *iNOS* in the infarcted hemisphere compared to the contralateral hemisphere (Figure 4, for full statistics see Supplementary Table S3). Interestingly, *TGF-β* expression was increased in the contralateral hemisphere by day-8 compared to sham, but drastically decreased in the infarcted side (Figure 4). K_V_1.3 inhibition with early and late PAP-1 or with the peptide ShK-223, reduced mRNA expression of *IL-1β*, *IL-6*, *TNF-α, COX-2* and *iNOS* (Figure 4) and increased expression of *TGF-β* in the infarcted hemisphere. Expression of *IFN-γ* was only significantly reduced by early PAP-1 treatment, while CD68 expression was not significantly affected by K_V_1.3 blocker treatment.

In contrast to previously published qPCR data from isolated microglia, whole brain tissue expression analysis of the microglial ion channels (*P2X4*, *P2X7*, *K*_*V*_ *1.3* and *K*_*Ca*_ *3.1*) was not very informative on day-8 (Figure 4). While there were trends towards increased mRNA expression with MCAO and decreased mRNA with treatment, only early PAP-1 treatment significantly decreased K_V_1.3 mRNA expression compared to vehicle treatment (Figure 4).

### 3.4 PAP-1 does not interfere with tPA *in-vitro*

**FIGURE 5:**
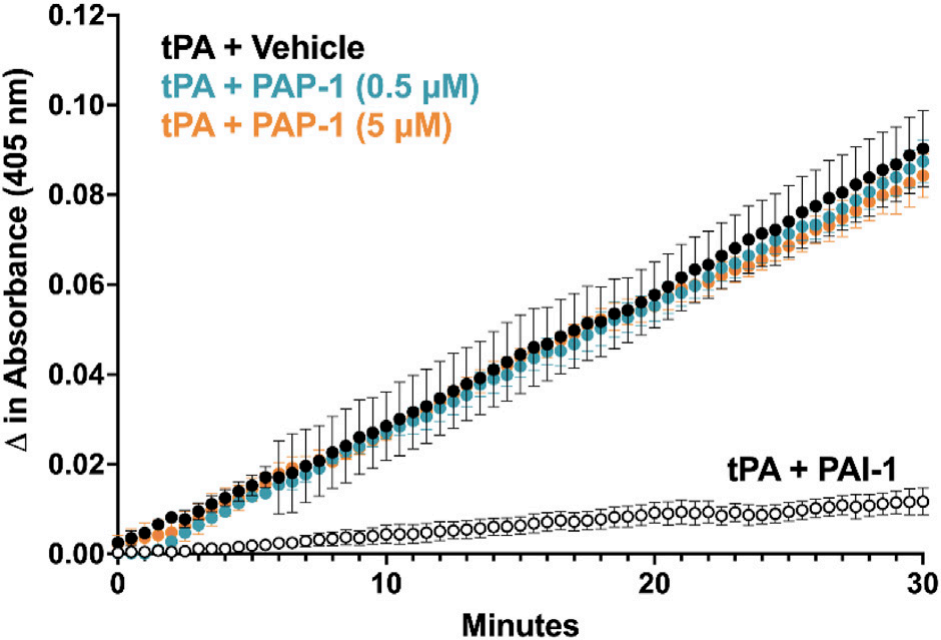
PAP-1 does not interfere with tPA activity *in vitro*. PAP-1 at 0.5 μM and 5 μM does not inhibit tPA activity as measured by increases in absorbance at 405 nm of a chromogenic tPA substrate. Each data point shown is the average of three independent experimental runs ±S.E.M. Each reaction was performed in triplicate wells.

In clinical use, K_V_1.3 inhibitors might not only be employed in combination with mechanical thrombectomy but also together with, or shortly after tPA infusion. We, therefore, tested whether PAP-1 affects the proteolytic function of tPA (Figure 5). While the positive control, plasminogen activator inhibitor-1 (PAI-1) strongly inhibited the enzymatic activity of tPA in a chromogenic assay, PAP-1, at 0.5 μM and 5 μM, had no effect on tPA activity during the 30 min assay suggesting that it is unlikely to affect its fibrinolytic activity.

## 4 Discussion

Since K_V_1.3 was first discovered in T-cells in 1984 (DeCoursey et al., 1984), the channel has been studied as a potential target for immunosuppression and immunomodulation in multiple sclerosis, autoimmune diabetes, rheumatoid arthritis, psoriasis and ulcerative colitis (Beeton et al., 2006; Kundu-Raychaudhuri et al., 2014; Unterweger et al., 2021). K_V_1.3 blockers, like ShK-peptides or the small molecules PAP-1 and DES1, work well in treating these T-cell mediated pathologies because K_V_1.3 is overexpressed on CCR7^-^ effector memory T cells (Beeton et al., 2006), which are a subset of T-cells that home to sites of inflammation, secrete pro-inflammatory cytokines, and display immediate effector functions (Sallusto et al., 1999). More recently, K_V_1.3 has been investigated as a target for inflammation in the central nervous system where K_V_1.3 expression has been described on activated, “M1-like” or disease-associated microglia in human brain or in rodent models of Alzheimer’s disease (Maezawa et al., 2018; Ramesha et al., 2021), multiple sclerosis (Rus et al., 2005), Parkinson’s disease (Sarkar et al., 2020), and ischemic stroke (Chen et al., 2018). Network analysis of transcriptomic datasets revealed that K_V_1.3 is part of a pro-inflammatory microglial gene signature in neurodegenerative disease models (Rangaraju et al., 2017; Ramesha et al., 2021). The physiological function of K_V_1.3 in T cells and microglia is similar; this voltage-gated potassium channel regulates membrane potential and controls store-operated calcium influx through CRAC (calcium release activated calcium) channels and downstream signaling events such as activation and nuclear translocation of NF-kB and NFAT (Feske et al., 2015; Fomina et al., 2021). In microglia, K_V_1.3 also plays a crucial role in enabling these cells to resist depolarizations produced by P2X4 receptor activation by the danger signal ATP (Nguyen et al., 2020). In assays with cultured microglia or organotypic hippocampal slices, K_V_1.3 blockers reduce expression of iNOS and COX-2 and production of inflammatory cytokines and NO following stimulation with LPS, amyloid-β, α-synuclein or ischemia/hypoxia (Chen et al., 2018; Maezawa et al., 2018; Sarkar et al., 2020). K_V_1.3 blockers, which are generally well tolerated, and do not affect the ability of rodents to clear infections or of rhesus macaques to develop protective vaccine responses (Beeton et al., 2006; Pereira et al., 2007), therefore constitute attractive therapeutic candidates for immunocytoprotection in ischemic stroke.

In our efforts to translate K_V_1.3 into a therapeutic target for ischemic stroke, we initially performed proof-of-concept experiments in young male mice, where we characterized the time course of K_V_1.3 expression on microglia after MCAO and demonstrated that PAP-1 treatment started at 12 h after reperfusion decreased infarct size and improved neurological deficits after 1 week (Chen et al., 2018). We subsequently evaluated whether K_V_1.3 inhibition would also be beneficial in females and aged animals. At the target level, there are no sex differences; microglia from both male and female mice express similar levels of K_V_1.3 in culture or after acute isolation out of the infarcted area following MCAO (Nguyen et al., 2020; Chen et al., 2021). In treatment experiments, PAP-1 started at 12 h after reperfusion benefited young and aged mice of both sexes, but was particularly effective in 20-month-old animals (Chen et al., 2021), presumably because of the increasing T-cell contribution to stroke pathology due to a process that has been termed “inflammaging” (Carrasco et al., 2021), and which is characterized by a shrinking of the naïve T cell compartment and an expansion of the tissue homing effector memory T-cell pool. In keeping with this hypothesis, we observed that both treatment with PAP-1 or genetic K_V_1.3 deletion, not only reduced microglia/macrophage activation as measured by Iba-1 pixel density but also very effectively reduced T cell infiltration into the infarcted hemisphere in aged animals (Chen et al., 2021).

In this present study, we focus on exploring the treatment window for K_V_1.3 inhibition and the treatment modalities. Specifically, we wanted to determine whether K_V_1.3 blockers for the treatment of ischemic stroke need to be brain-penetrant small molecules or whether biologics would also be effective. While our small molecule PAP-1 penetrates well into the brain and reaches equal concentrations in brain tissue and - plasma (C_brain_/C_plasma_ = 1.1) (Beeton et al., 2006), positively charged ShK peptides are only minimally brain penetrant based on studies with radiolabeled peptides that showed 0.05% of the total injected radioactivity in the brain (Tarcha et al., 2012). To answer this question about the treatment modality, we decided to directly compare PAP-1 and the peptide ShK-223 in MCAO in male rats with treatment started 2 h after reperfusion, a time point that would simulate administration relatively early after mechanical thrombectomy. In this setting, where both PAP-1 and ShK-223 were given at doses at which they had previously been effective in stroke or EAE in rats (Tarcha et al., 2012; Chen et al., 2018), the peptide ShK-223 failed to significantly reduce T2-weighted infarct area or improve neurological deficit scoring on day-8, while the small molecule PAP-1 improved both parameters. When delaying the start of PAP-1 administration, we found that even if treatment is started at 3 days after reperfusion, PAP-1 is still effective at reducing infarct area and improving neurological deficit on day-8, presumably by salvaging the inflammatory penumbra.

One very interesting observation from our study is that both the peptide ShK-223 and early or late administration of the small molecule PAP-1 were similarly effective at reducing expression of *IL-1β*, *IL-6*, *TNF-α, COX-2* and *iNOS* in the infarcted hemisphere on day-8. Yet only PAP-1 treatment resulted in a significant reduction in infarction and an improvement in neurological deficits. Why is there such a mismatch between inflammatory marker expression and functional outcomes? Our interpretation of these findings is that a large proportion of the mRNA in the infarcted tissue on day-8 originates from infiltrating peripheral immune cells that have migrated to and proliferated in the infarcted tissue. These cells would have been exposed to ShK-223 in the periphery and susceptible to the effects of this membrane-impermeable peptide. A limitation of our study in this respect is that we used all animals for qPCR and did not directly compare the extent of immune cell infiltration/activation by immunohistochemistry or flow cytometry between ShK-223, early PAP-1 and late PAP-1 treatment. Another explanation for the lack of effect of the peptide on functional outcomes is, that it was not able to access the infarcted tissue sufficiently. Opening of the blood brain barrier (BBB) in tMCAO has been reported to be biphasic, once at 6 h and once at 72 h, with no Evans blue or fluorescein extravasation occurring at 24 or 48 h (Hone et al., 2018). However, even under conditions where the brain is inflamed, and the BBB confirmed to be compromised, only very small amounts of ShK-sized peptides (4 kDa) penetrate into the brain as a recently published PET imaging study using HsTX1 labeled with a long-lived ^64^Cu-isotope demonstrated (Reddiar et al., 2022). Similar to ShK-223, HsTX1 [R14A] is a 34-residue, C-terminally amidated peptide cross-linked by four disulfide bridges, that inhibits K_V_1.3 with picomolar affinity. When LPS treatment was used to open-up the blood brain barrier, brain uptake of the ^64^Cu-labeled HsTX1 [R14A] only increased from less than 0.1 SUV (standard uptake value) to 0.25 SUV compared to SUVs of 40-95 in the kidney, demonstrating very limited bioavailability in the brain even with a compromised BBB (Reddiar et al., 2022). Similar results had been obtained with an LS-MS/MS assay (Reddiar et al., 2021). While no HsTX1 [R14A] was detectable in the brains of normal mice, brain concentrations reached 2% of plasma concentrations following treatment with a high dose of systemic LPS. The small molecule PAP-1, in contrast, is highly brain penetrant and provides solid brain exposure with total concentrations in the micromolar range and free brain concentrations providing IC_50_-IC_90_ coverage of K_V_1.3 for at least 8 h after administration in mice or rats (Beeton et al., 2006; Chen et al., 2018; Maezawa et al., 2018). One major conclusion from our study is, therefore, that K_V_1.3 inhibitors used for immunocytoprotection after ischemic stroke need to be brain penetrant to prevent inflammatory lesion expansion. These findings are similar to observations made with targeting P2X7 for stroke. P2X7 inhibiting nanobodies can only occupy microglial P2X7 receptor channels and reduce infarction following intracerebroventricular delivery but not after intravenous administration (Pinto-Espinoza et al., 2022; Wilmes et al., 2022), indicating insufficient crossing of the blood-brain barrier by the nanobodies.

Our other major finding in this study is that K_V_1.3 inhibition with PAP-1 still improves MCAO outcomes when administration is started as late as 3 days after reperfusion. This is an exciting observation that is in keeping with the time course of microglia/macrophages activation (Campanella et al., 2002; Hu et al., 2012) and K_V_1.3 expression after ischemic stroke, which starts to significantly increase between 2–5 days and peaks around day-8 after MCAO (Chen et al., 2018). From a therapeutic perspective, these findings suggest that K_V_1.3 inhibition has a wide treatment window and could be a useful, adjunctive pharmacological treatment that could be administered either together with EVT or fibrinolytic drugs or up to days later to reduce inflammation in the penumbra and prevent reperfusion injury.

## Data Availability

The original contributions presented in the study are included in the article/Supplementary Material, further inquiries can be directed to the corresponding author.
