# Supplementary material for: Immunocytoprotection after reperfusion with Kv1.3 inhibitors has an extended treatment window for ischemic stroke
Source: Front Pharmacol. 2023 Apr 25;14:1190476. doi: 10.3389/fphar.2023.1190476 (PMC10166874; doi:10.3389/fphar.2023.1190476)
Supplement: Supplementary file 1 [file DataSheet1.PDF]

Supplementary Material

Immunocytoprotection after Reperfusion with Kv1.3 Inhibitors has an Extended Treatment Window for Ischemic Stroke

Ruth D. Lee[1§], Yi-Je Chen[1,2§], Latika Singh[1], Hai M. Nguyen[1], Heike Wulff[1]*

*** Correspondence:** Heike Wulff: hwulff@ucdavis.edu

1 Supplementary Figures and Tables

1 Supplemental Figure showing a comparison of the two vehicles, miglyol and PBS, to each other and of ShK-223 and PAP-1 to a combination of the two vehicle groups.

1 Supplemental Figure showing Kv1.3 expression on acutely isolated CD11b$^+$ cells from the infarcted hemisphere on day-8 after MCAO in rats confirming previous findings in mice.

3 Supplementary Tables showing the sequences of qPCR primers and the full statistic for the qPCR experiment in Figure 4.

1.1 Supplementary Figures

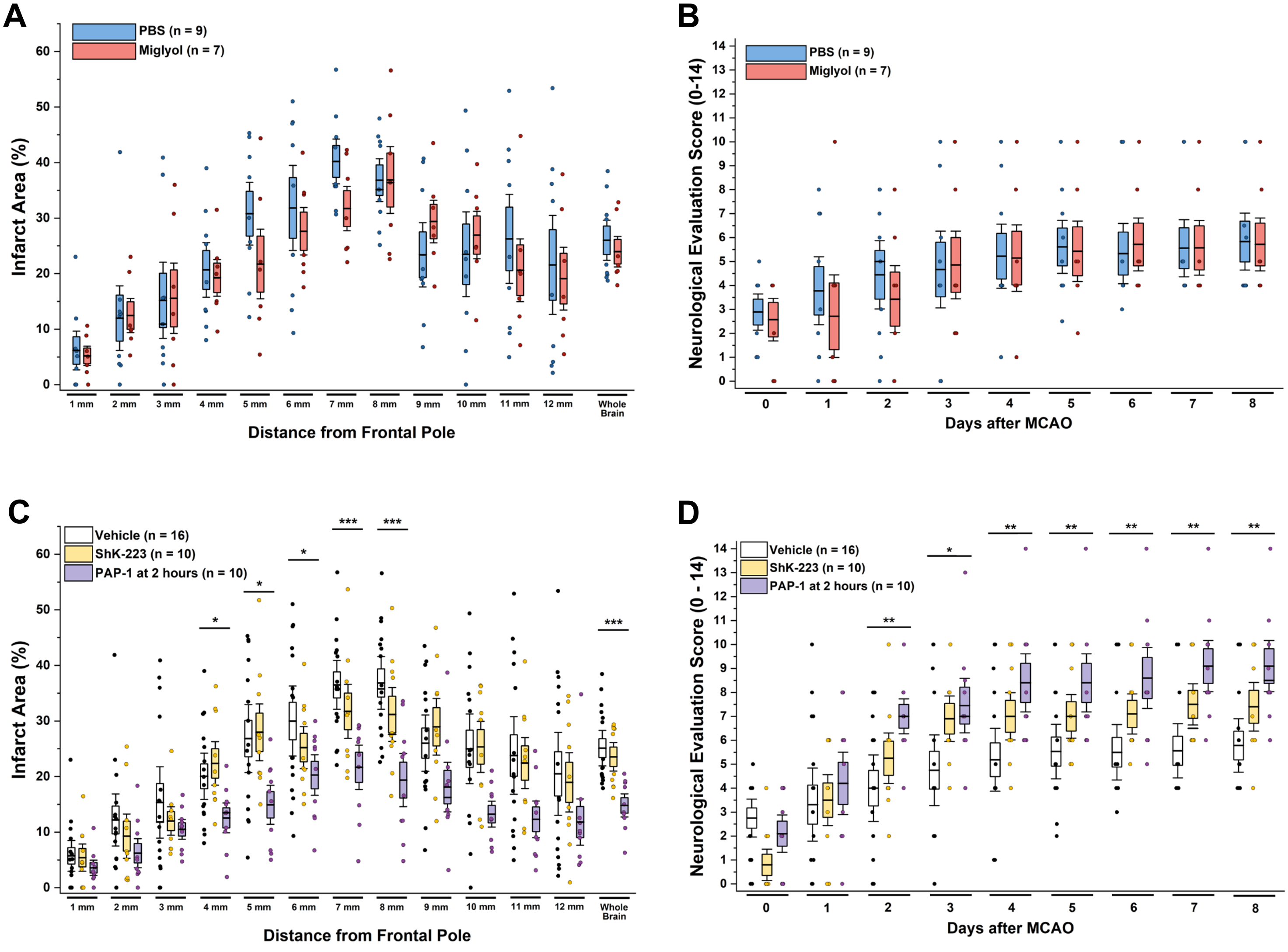

Supplementary Figure 1. The Kv1.3 blocker PAP-1 but not the peptide ShK-223 reduces infarction and improves neurological deficit in adult male rats. (A) Infarct area and **(B)** neurological deficit score in PBS (n = 9) compared to miglyol (n = 7) treated male Wistar rats demonstrating that there is no difference between the two vehicles. **(C)** Infarct and **(D)** neurological deficit score in vehicle treated male Wistar rats (n = 16) compared to PAP-1 (40 mg/kg; n = 10, P = 0.0004 for infarct, P = 0.003 for NES on day-8) treated rats and to ShK-223 (100 mg/kg; n = 10, P = 0.79103 for infarct, P = 0.2040 for NES on day-8) treated male Wistar rats. Data are shown as whisker plots with data from individual animals overlayed as scatter. The boxes show mean ± S.E.M, the whiskers show confidence intervals.

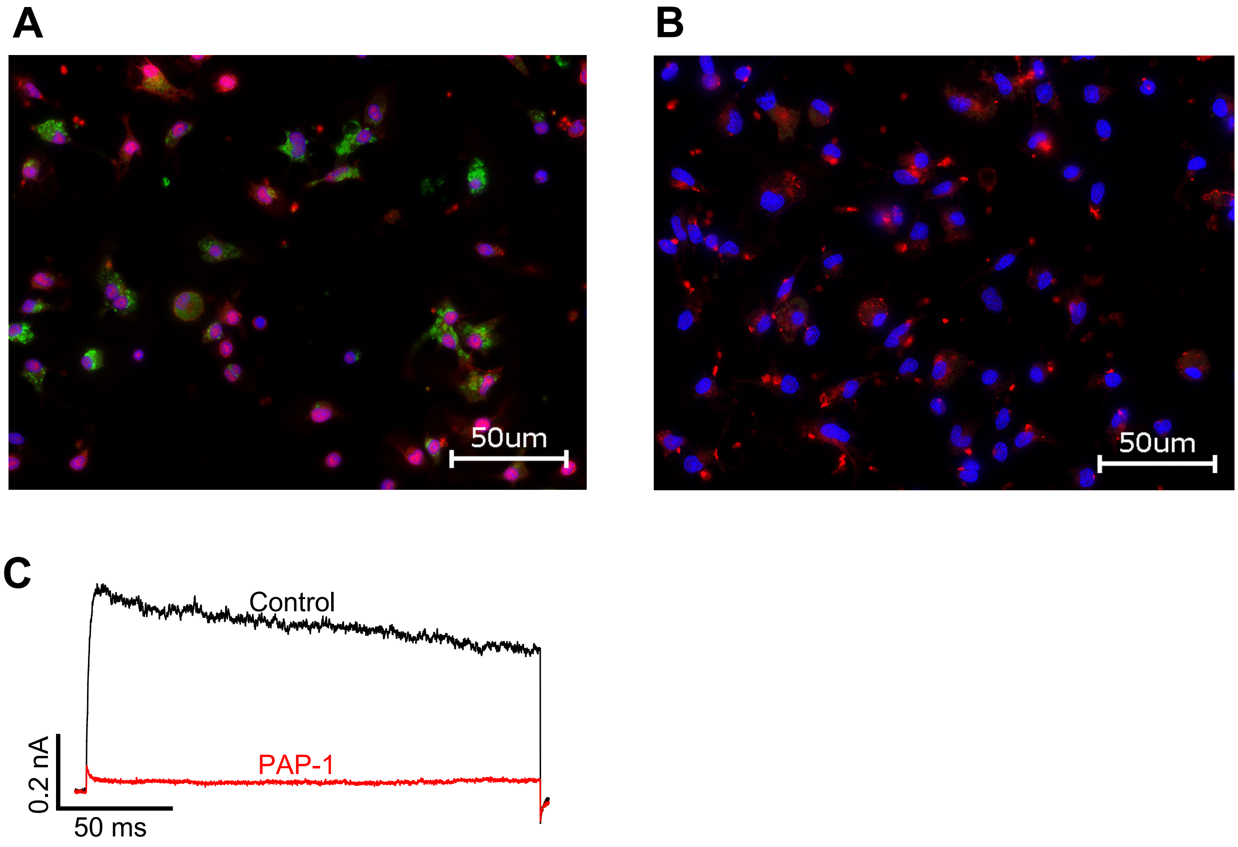

Supplementary Figure 2. Acutely isolated $CD11b^+$ cells from the infarcted hemisphere on day-8 after MCAO in rats express K_V1.3 as determined by immunofluorescence and electrophysiology. Brain tissue was dissociated enzymatically with a Neural Tissue Dissociation Kit (Miltenyi Biotec), microglia isolated using anti-CD11b magnetic beads (Miltenyi Biotec) as described previously (Chen et al., 2018) and plated on poly-L-lysine coated coverslips, permeabilized with 4% buffered formalin and then stained. **(A)** All isolated cells are positive for Iba-1 (rabbit anti-Iba1, Cat#019-19741,Wako 1:3000; secondary antibody is Alexa Fluo®546 goat anti-rabbit IgG(H+L), Cat# A11010, Life Technologies,1:1000) and many activated cells are positive for ED1 (= CD68; mouse anti-rat-CD68; Cat# MCA341R, Bio-Rad. 1:1000; secondary antibody is Alexa Fluo®488 donkey anti-mouse IgG(H+L), Cat# A21202, Life Technologies,1:1000). **(B)** Many activated cells stain for K_V1.3 (rabbit anti-K_v1.3, Cat# APC101, Alomone Labs 1:1000; secondary antibody is Alexa Fluo®546 goat Anti-rabbit IgG(H+L), Cat# A11010, Life Technologies,1:1000). **(C)** K_V currents were elicited by a 200-ms step pulse from -80 mV to + 40 mV as described (Chen et al., 2018). The current is blocked completely by 1 µM of PAP-1.

1.2 Supplementary Tables

Supplementary Table 1

**Gene**	**F Primer**	**R Primer**
il1b	CACCTCTCAAGCAGAGCACAG	GGGTTCCATGGTGAAGTCAAC
il6	TCCTACCCCAACTTCCAATGCTC	TTGGATGGTCTTGGTCCTTAGCC
cd68	CTCATCATTGGCCTGGTCCT	GTTGATTGTCGTCTGCGGG
cox2	TGTATGCTACCATCTGGCTTCGG	GTTTGGAACAGTCGCTCGTCATC
ifng	ATGAGTGCTACACGCCGCGTCTTGG	GAGTTCATTGACAGCTTTGTGCTGG
inos	GACCAGAAACTGTCTCACCTG	CGAACATCGAACGTCTCACA
tnfa	AAATGGGCTCCCTCTCATCAGTTC	TCTGCTTGGTGGTTTGCTACGAC
tgfb	CAAAGACATCACACACAGTA	GGTGTTGAGCCCTTTCCAGG
*il2**	CATGTACAGCATGCAGCTCGCATCC	CCACCACAGTTGCTGGCTCATCATC
*il4**	TGCACCGAGATGTTTGTACC	GGATGCTTTTTAGGCTTTCC
*il10**	GCAGGACTTTAAGGGTTACTTGG	GGGGAGAAATCGATGACAGC
*il17a**	CTACCTCAACCGTTCCACT	TTCTCAGGCTCCCTCTTC

*These primers did not work in our hands.

**Gene**	**Biorad Unique Assay ID**
actb	qRnoCID0056984
hmbs	qRnoCED0057013
ywhaz	qRnoCID0056990
kcnn4	qRnoCID0003361
kcna3	qRnoCED0018850
p2rx4	qRnoCID0003238
p2rx7	qRnoCID0007779

Supplementary Table 2

Housekeeping Gene plate from BioRad

Gene	PrimePCR Unique Assay ID
actb	qRnoCID0056984
b2m	qRnoCED0056999
camlg	qRnoCED0009273
cd163	qRnoCID0008321
gapdh	qRnoCID0057018
hmbs	qRnoCED0057013
hprt1	qRnoCED0057020
pgk1	qRnoCED0002588
polr2a	qRnoCED0007537
ppib	qRnoCED0006997
ppox	qRnoCED0012992
rpl13a	qRnoCED0056993
rps13	qRnoCED0002931
rps18	qRnoCED0003920
sdha	qRnoCID0057011
tubb5	qRnoCED0053789
ywhaz	qRnoCID0056990

Supplementary Table 3: Statistics for qPCR

Treatment groups	Samples
Sham	6
Vehicle Contralateral	12
Vehicle Ipsilateral	10
PAP-1 (2 hr) Ipsilateral	9
PAP-1 (3 days) Ipsilateral	11
ShK-223 (2 hr) Ipsilateral	12

il-1b

Dunnett's multiple comparisons test	Mean Diff.	95.00% CI of diff.	P-Value	Summary
Ipsilateral + Vehicle vs. Sham	2.331	0.9230 to 3.739	<0.001	***
Ipsilateral + Vehicle vs. Contralateral + Vehicle	2.19	1.004 to 3.377	<0.001	***
Ipsilateral + Vehicle vs. PAP-1 (2 hour)	2.63	1.357 to 3.904	<0.001	***
Ipsilateral + Vehicle vs. PAP-1 (3 day)	2.412	1.201 to 3.623	<0.001	***
Ipsilateral + Vehicle vs. ShK	1.84	0.6010 to 3.080	0.001	**

il-6

Dunnett's multiple comparisons test	Mean Diff.	95.00% CI of diff.	P-Value	Summary
Ipsilateral + Vehicle vs. Sham	5.436	1.851 to 9.022	0.001	**
Ipsilateral + Vehicle vs. Contralateral + Vehicle	4.524	1.466 to 7.583	0.001	**
Ipsilateral + Vehicle vs. PAP-1 (2 hour)	5.261	1.992 to 8.531	<0.001	***
Ipsilateral + Vehicle vs. PAP-1 (3 day)	5.346	2.229 to 8.463	<0.001	***
Ipsilateral + Vehicle vs. ShK	4.621	1.434 to 7.808	0.002	**

tnfa

Dunnett's multiple comparisons test	Mean Diff.	95.00% CI of diff.	P-Value	Summary
Ipsilateral + Vehicle vs. Sham	2.655	1.582 to 3.727	<0.001	***
Ipsilateral + Vehicle vs. Contralateral + Vehicle	2.431	1.500 to 3.363	<0.001	***
Ipsilateral + Vehicle vs. PAP-1 (2 hour)	1.809	0.8295 to 2.789	<0.001	***
Ipsilateral + Vehicle vs. PAP-1 (3 day)	2.571	1.617 to 3.524	<0.001	***
Ipsilateral + Vehicle vs. ShK	2.195	1.241 to 3.148	<0.001	***

ifng

Dunnett's multiple comparisons test	Mean Diff.	95.00% CI of diff.	P-Value	Summary
Ipsilateral + Vehicle vs. Sham	3.376	1.032 to 5.720	0.002	**
Ipsilateral + Vehicle vs. Contralateral + Vehicle	2.412	0.4320 to 4.392	0.01	*
Ipsilateral + Vehicle vs. PAP-1 (2 hour)	3.512	1.388 to 5.637	<0.001	***
Ipsilateral + Vehicle vs. PAP-1 (3 day)	1.832	-0.1888 to 3.852	0.09	ns
Ipsilateral + Vehicle vs. ShK	1.306	-0.7622 to 3.374	0.38	ns

Cd68

Dunnett's multiple comparisons test	Mean Diff.	95.00% CI of diff.	P-Value	Summary
Ipsilateral + Vehicle vs. Sham	38.4	9.213 to 67.59	0.006	**
Ipsilateral + Vehicle vs. Contralateral + Vehicle	37.15	10.90 to 63.40	0.002	**
Ipsilateral + Vehicle vs. PAP-1 (2 hour)	1.615	-26.44 to 29.68	>0.99	ns
Ipsilateral + Vehicle vs. PAP-1 (3 day)	18.67	-8.676 to 46.02	0.3	ns
Ipsilateral + Vehicle vs. ShK	18.45	-8.901 to 45.80	0.31	ns

cox2

Dunnett's multiple comparisons test	Mean Diff.	95.00% CI of diff.	P-Value	Summary
Ipsilateral + Vehicle vs. Sham	3.581	2.477 to 4.684	<0.001	***
Ipsilateral + Vehicle vs. Contralateral + Vehicle	3.107	2.199 to 4.015	<0.001	***
Ipsilateral + Vehicle vs. PAP-1 (2 hour)	3.688	2.713 to 4.662	<0.001	***
Ipsilateral + Vehicle vs. PAP-1 (3 day)	3.662	2.736 to 4.589	<0.001	***
Ipsilateral + Vehicle vs. ShK	3.997	3.048 to 4.945	<0.001	***

inos

Dunnett's multiple comparisons test	Mean Diff.	95.00% CI of diff.	P-Value	Summary
Ipsilateral + Vehicle vs. Sham	3.634	1.080 to 6.188	0.003	**
Ipsilateral + Vehicle vs. Contralateral + Vehicle	2.684	0.5077 to 4.860	0.01	**
Ipsilateral + Vehicle vs. PAP-1 (2 hour)	4.148	1.821 to 6.475	<0.001	***
Ipsilateral + Vehicle vs. PAP-1 (3 day)	3.541	1.322 to 5.759	<0.001	***
Ipsilateral + Vehicle vs. ShK	3.097	0.8289 to 5.364	0.003	**

tgfb

Dunnett's multiple comparisons test	Mean Diff.	95.00% CI of diff.	P-Value	Summary
Ipsilateral + Vehicle vs. Sham	-0.6499	-3.937 to 2.638	0.98	ns
Ipsilateral + Vehicle vs. Contralateral + Vehicle	-1.869	-4.955 to 1.217	0.42	ns
Ipsilateral + Vehicle vs. PAP-1 (2 hour)	-4.63	-7.876 to -1.385	0.002	**
Ipsilateral + Vehicle vs. PAP-1 (3 day)	-3.716	-6.875 to -0.5573	0.01	*
Ipsilateral + Vehicle vs. ShK	-3.764	-6.922 to -0.6049	0.01	*

p2x4

Dunnett's multiple comparisons test	Mean Diff.	95.00% CI of diff.	P-Value	Summary
Ipsilateral + Vehicle vs. Sham	1.166	-0.4555 to 2.787	0.24	ns
Ipsilateral + Vehicle vs. Contralateral + Vehicle	0.9141	-0.4772 to 2.305	0.34	ns
Ipsilateral + Vehicle vs. PAP-1 (2 hour)	0.1509	-1.342 to 1.644	>0.99	ns
Ipsilateral + Vehicle vs. PAP-1 (3 day)	0.6475	-0.8057 to 2.101	0.72	ns
Ipsilateral + Vehicle vs. ShK	0.5527	-0.9005 to 2.006	0.83	ns

p2x7

Dunnett's multiple comparisons test	Mean Diff.	95.00% CI of diff.	P-Value	Summary
Ipsilateral + Vehicle vs. Sham	0.09769	-1.014 to 1.210	>0.99	ns
Ipsilateral + Vehicle vs. Contralateral + Vehicle	0.06285	-0.9863 to 1.112	>0.99	ns
Ipsilateral + Vehicle vs. PAP-1 (2 hour)	0.584	-0.5783 to 1.746	0.61	ns
Ipsilateral + Vehicle vs. PAP-1 (3 day)	-0.5905	-1.661 to 0.4801	0.52	ns
Ipsilateral + Vehicle vs. ShK	-0.2983	-1.394 to 0.7975	0.96	ns

kcnn4

Dunnett's multiple comparisons test	Mean Diff.	95.00% CI of diff.	P-Value	Summary
Ipsilateral + Vehicle vs. Sham	6.439	0.3250 to 12.55	0.04	*
Ipsilateral + Vehicle vs. Contralateral + Vehicle	5.896	0.6723 to 11.12	0.02	*
Ipsilateral + Vehicle vs. PAP-1 (2 hour)	2.393	-3.394 to 8.180	0.78	ns
Ipsilateral + Vehicle vs. PAP-1 (3 day)	1.007	-4.449 to 6.463	>0.99	ns
Ipsilateral + Vehicle vs. ShK	1.175	-4.281 to 6.631	0.99	ns

kcna3

Dunnett's multiple comparisons test	Mean Diff.	95.00% CI of diff.	P-Value	Summary
Ipsilateral + Vehicle vs. Sham	0.6518	-0.3841 to 1.688	0.35	ns
Ipsilateral + Vehicle vs. Contralateral + Vehicle	0.6974	-0.1631 to 1.558	0.16	ns
Ipsilateral + Vehicle vs. PAP-1 (2 hour)	0.9841	0.06075 to 1.907	0.03	*
Ipsilateral + Vehicle vs. PAP-1 (3 day)	0.7824	-0.09568 to 1.660	0.1	ns
Ipsilateral + Vehicle vs. ShK	0.5407	-0.3581 to 1.439	0.43	ns
